# Expressions of IFN-γ and IL-4 before and after Treatment of Lupus Nephritis with Traditional Chinese Medicine Combined with Cyclophosphamide and Their Values for Efficacy Prediction and Evaluation

**Published:** 2020-05

**Authors:** Yan JIANG, Qichang ZHANG, Haoyu WANG, Dabei TANG, Yan ZHANG, Yimo, ZHANG, Li YU

**Affiliations:** 1. Department of Traditional Chinese Medicine, The First Hospital of Qiqihar City, Qiqihar City 161000, China; 2. Department of Basic Science of Traditional Chinese Medicine, Heilongjiang University of Chinese Medicine, Harbin City 150040, China; 3. Department of Oncology, The Third Affiliated Hospital of Harbin Medical University, Harbin City 150040, China; 4. Department of Nephrology, The Second Affiliated Hospital of Qiqihar Medical College, Qiqihar City 161006, China; 5. Department of Nephrology, Qiqihar Traditional Chinese Medicine Hospital, Qiqihar City 161006, China

**Keywords:** Interleukin-4, Traditional Chinese medicine, Cyclophosphamide, Lupus nephritis

## Abstract

**Background:**

To explore IFN-γ (interferon-γ) and IL-4 (interleukin-4) expressions before and after the treatment of LN (lupus nephritis) and their values for efficacy prediction and evaluation.

**Methods:**

Altogether 107 patients with LN treated in the First Hospital of Qiqihaer City, Qiqihar, China from March 2017 to September 2018 were enrolled. Sixty-two patients were treated with cyclophosphamide and prednisolone (control group), while another 45 patients were treated with Qing Shen Fang based on the control group (observation group). Their clinical efficacy and changes in immune indices after treatment were observed.

**Results:**

Compared with those in the control group, clinical efficacy, IFN-γ, IL-4, hemoglobin, complements C3 and C4, ESR (erythrocyte sedimentation rate), serum IgG, SLEDAI (Systemic Lupus Erythematosus Disease Activity Index) score, and TCMSSS (Traditional Chinese Medicine Syndrome Score Scale) score were significantly improved after treatment in the study group. Based on the observation, IFN-γ and IL-4 could be used as potential indicators for evaluating clinical efficacy.

**Conclusion:**

The combination of cyclophosphamide, prednisolone, and Qing Shen Fang improves conditions of patients with LN and significantly reduces their IFN-γ and IL-4 levels in serum. IFN-γ and IL-4 can be used as potential indicators for the efficacy prediction and evaluation of the disease.

## Introduction

Systemic lupus erythematosus (SLE) is an auto-immune disease involving multiple systems and organs. After its onset, lymphocytes in the body significantly proliferate and produce a large number of antibodies and immune complexes that are deposited in various organs, of which kidney is one of the most common ones involved (1, 2). Almost all patients with SLE suffer from renal injury, which is clinically called lupus nephritis (LN). About 20% of patients initially diagnosed with LN progress to those with end-stage renal disease within 10 years, which eventually leads to patient deaths (3). Therefore, effective and timely treatment is important for improving the patients’ conditions.

Currently, patients with LN are mainly treated by corticosteroids and immunosuppressive agents, but the proportion of progressing to end-stage renal disease remains around 10%-20%, which has not been significantly reduced (4). Although cyclophosphamide as the first choice for the treatment of LN can significantly improve the patients’ conditions, its effective rate is 70%–80% and it has large adverse drug reactions (5, 6).

Compared with western medicine, Chinese traditional medicine puts into effect slower, causes fewer toxic and side effects, and has a lower recurrence rate (7). Qing Shen Fang is effective for treating LN, but whether its combination with cyclophosphamide can improve the patients’ conditions remains unclear. There are currently few standards for the efficacy prediction and prognostic evaluation of the disease. IFN-γ (interferon-γ) belongs to Th1 cells that mediate cellular immunity, and IL-4 (interleukin-4) belongs to Th2 cells that activate B cells to produce antibodies such as immunoglobulin (8). A recent study shows that their expressions significantly change after the onset of LN (9). However, whether the expressions can be used as potential predictive markers for the efficacy in the treatment of LN has been rarely studied.

Therefore, the expressions of IFN-γ and IL-4 before and after the treatment of LN with traditional Chinese medicine combined with cyclophosphamide and their values for efficacy prediction and evaluation were explored in this study, in order to provide references for the treatment and efficacy prediction of LN.

## Materials and Methods

### Patient data

A total of 107 patients with LN treated in the First Hospital of Qiqihaer City, Qiqihar, China from March 2017 to September 2018 were enrolled in this study. Among them, 62 patients were treated with cyclophosphamide combined with prednisolone (the control group), while another 45 patients were treated with Qing Shen Fang on the basis of the control group (the observation group).

This study was approved by the Medical Ethics Committee of our hospital.

### Inclusion and exclusion criteria

Inclusion criteria were as follows: patients who met the diagnostic criteria for SLE from the American College of Rheumatology in 1997 (10); patients with hematuria, proteinuria, cylindruria, and renal function impairment; patients confirmed with renal injury by pathological biopsy; patients with a SLEDAI (Systemic Lupus Erythematosus Disease Activity Index) score ≤10 points; patients with complete clinical data; patients who and whose families had been informed and signed an informed consent form.

Exclusion criteria were as follows: patients with advanced LN; patients complicated with tumors; patients with infections caused by other factors; patients with heart, liver, brain, blood, and endocrine dysfunction; patients with cognitive defects; lactating or pregnant patients.

### Main drugs and kits

Cyclophosphamide (Jiangsu Suncadia Biopharmaceuticals Co., Ltd., China, H32024654); prednisolone (Zhejiang Xianju Pharmaceutical Co., Ltd., China, H33021225); IFN-γ and IL-4 ELISA kits (Beyotime Biotechnology, Shanghai, China, PI511, PI618). Chinese herbs in Qing Shen Fang were prepared by our hospital and provided by our pharmacy.

### Therapeutic schemes

Patients in the control and observation groups were treated with cyclophosphamide and prednisolone. Schemes were as follows: the patients were administrated with prednisolone (1 mg/kg), once/day for 6 weeks. After that, the dosage was reduced to 5–10 mg/d for lifetime. The patients were intravenously dripped with cyclophosphamide (0.5–1.0 g/m^2^) which was diluted with normal saline (250 mL) or 5% glucose solution, once/month for the first 6 months and then once every 3 months. They were treated for a total of 2 years. On this basis, patients in the observation group were additionally treated with Qing Shen Fang (prescription composition is shown in Table 1), 1 agent/day. The drugs were decocted with water to obtain medicine juices, which were warmed and taken before breakfast and dinner. The clinical efficacy was compared between the two groups after treatment for 16 weeks (after treatment).

**Table 1:** Prescription composition

***Chinese name***	***English name***	***Dosage (g)***
Qinghao	Sweet wormwood herb	18
Shengshigao	Gypsum	25
Biejia	Turtle carapace	10
Shudi	Prepared rhizome of rehmannia	20
Zhimu	Common anemarrhena	15
Huangqin	Baical skullcap root	10
Huanglian	Chinese goldthread rhizome	5
Danshen	Danshen root	15
Chishao	Red peony root	10
Shuiniujiao	Buffalo horn	20
Xuanshen	Figwort root	15
Baihuasheshecao	Spreading hedyotis herb	15
Zhizi	Common gardenia fruit	15
Danpi	Cortex moutan	15
Shanzhuyu	Common macrocarpium fruit	15
Gancao	Liquorice root	6

### Sample collection and detection

The patients’ peripheral venous blood (5 mL) was extracted before and after treatment, allowed to stand for 30 min and then centrifuged at 3000 rpm for 10min, so as to obtain serum. The expressions of IFN-γ and IL-4 in the serum was detected using the ELISA kits, with specific steps conducted based in a previous study (11).

### Data collection

The expressions of hemoglobin, ESR (erythrocyte sedimentation rate), complement C3, complement C4, and serum IgG before and after treatment were collected by Laboratory Information System in the Laboratory Medicine.

### Observational indices

Main observational indices: The clinical efficacy in the two groups was evaluated, which referred to the 2002 edition of *Guiding Principles of Clinical Research on New Drugs of Chinese Medicines* (12) (More details are shown in Table 2). Patients in the two groups were compared in terms of the expressions of IFN-γ and IL-4, SLEDAI score, and TCMSSS (Traditional Chinese Medicine Syndrome Score Scale) score (13) before and after treatment. Secondary observational indices: The expressions of clinical indicators (hemoglobin, ESR, complement C3, complement C4, and serum IgG) before and after treatment were compared. According to the efficacy, patients were divided into the excellent efficacy group (Group A) and the poor efficacy group (Group B). IFN-γ and IL-4 before treatment were compared between the two groups. ROC (receiver operating characteristic) curves were plotted to analyze the predictive values of IFN-γ and IL-4 before treatment for efficacy in the treatment of LN. Pearson correlation test was used to analyze their correlations with clinical indicators, SLEDAI score, and TCMSSS score after treatment. Adverse reactions in the two groups were observed.

**Table 2:** Efficacy evaluation

***Efficacy classification***	***Evaluation criteria***
Remissive	The patients’ main clinical symptoms and signs basically disappeared, and their TCMSSS score reduced by more than 90%, with 24-hour urinary protein quantity <0.2g and normal renal function.
Markedly effective	The clinical symptoms and signs basically disappeared or disappeared, and the TCMSSS score reduced by more than 70%, with 24-hour urine protein quantity <1.0g and basically normal renal function.
Effective	The clinical symptoms and signs were improved, and the TCMSSS score reduced by more than 30%, with 24-hour urine protein quantity <3.5g and improved renal function compared with before treatment.
Ineffective	The clinical manifestations and laboratory indexes were not improved and they even deteriorated.

### Statistical analysis

In this study, GraphPad Prism 7 was used to plot figures. Count data were expressed by rate (%), analyzed by chi-square test. Ranked data were analyzed by non-parametric test and represented by Z. Measurement data were expressed by mean ± standard deviation (SD±meas), and paired *t* test was used for comparison before and after treatment within groups, while independent samples *t* test was used for comparison between groups, which was represented by *t*. Multivariate Logistic regression was used to analyze risk factors affecting efficacy. ROC curves were plotted to analyze the predictive values of IFN-γ and IL-4 before treatment for efficacy. Pearson correlation test was used to analyze the correlations of IFN-γ and IL-4 with hemoglobin, ESR, complement C3, complement C4, serum IgG, SLEDAI score, and TCMSSS score after treatment. When *P*<0.05, the difference was statistically significant.

## Results

### Clinical data

There were no statistically significant differences between the observation and control groups in gender, age, course of disease, body mass index (BMI), past medical history, history of smoking, history of alcoholism, and place of residence (Table 3).

**Table 3:** Baseline data

***Factors***		***Observation group (n=45)***	***Control group (n=62)***	***χ^2^/t value***	***P value***
Gender					
	Male	5 (11.00)	4 (6.00)	0.735	0.391
	Female	40 (89.00)	58 (94.00)		
Age (Years)		37.5±10.1	36.8±9.4	0.369	0.713
Course of disease (Months)		22.15±7.21	22.95±7.66	0.547	0.586
BMI (kg/m^2^)		22.41±1.72	22.05±1.84	1.027	0.307
Past medical history					
	Hypertension	9 (20.00)	14 (23.00)	0.103	0.748
	Diabetes	8 (18.00)	15 (24.00)	0.636	0.425
History of smoking					
	Yes	9 (20.00)	8 (13.00)	0.983	0.322
	No	36 (80.00)	54 (87.00)		
History of alcoholism					
	Yes	3 (7.00)	3 (4.84)	0.165	0.685
	No	42 (93.00)	59 (95.16)		
Place of residence					
	City	25 (56.00)	32 (52.00)	0.163	0.687
	Countryside	20 (44.00)	30 (48.00)		

### Comparison of efficacy after treatment

The results of rank sum test showed that the clinical efficacy in the observation group was better than that in the control group (*P*=0.020) (Table 4).

**Table 4:** Efficacy evaluation

***Groups***	***Remissive***	***Markedly effective***	***Effective***	***Ineffective***	***Z value***	**P *value***
Control group (n=62)	4 (6.45)	18 (29.03)	27 (43.55)	13 (20.97)	−2.328	0.020
Observation group (n=45)	8 (17.78)	18 (40.00)	13 (28.89)	6 (13.33)		

### Expressions of IFN-γ and IL-4 before and after treatment

According the comparison of the expressions of IFN-γ and IL-4, before treatment, there was no difference in the expressions between the control and observation groups (*P*_IFN-γ_=0.104, *P*_IL-4_=0.839). After treatment, the expressions in the two groups were significantly lower than those before treatment, and the difference in the observation group was greater than that in the control group (*P*_IFN-γ_=0.001, *P*_IL-4_=0.001) (Tables 5 and 6).

**Table 5:** Expression of IFN-γ before and after treatment

**Groups**	**IFN-γ (pg/mL)**		**t *value***	**P *value***	***Difference***
	***Before treatment***	***After treatment***			
Control group (n=62)	22.07±2.84	18.38±2.87	106.918	<0.001	3.80±2.07
Observation group (n=45)	21.19±2.63	16.59±2.50	111.033	<0.001	4.69±2.05
*t* value	1.639	3.365			2.208
*P* value	0.104	0.001			0.029

**Table 6:** Expression of IL-4 before and after treatment

**Groups**	***IL-4 (pg/mL)***		**t *value***	**P *value***	***Difference***
	***Before treatment***	***After treatment***			
Control group (n=62)	55.00±7.32	47.84±7.04	97.561	<0.001	7.37±4.41
Observation group (n=45)	56.27±8.23	43.40±5.75	117.226	<0.001	13.38±5.52
*t* value	0.403	3.468			6.036
*P* value	0.839	0.001			<0.001

### Comparison of clinical indicators, SLEDAI score, and TCMSSS score before and after treatment

Before treatment, there were no significant differences between the control and observation groups in the clinical indicators, SLEDAI score, and TCMSSS score. After treatment, hemoglobin, complement C3, and complement C4 in the two groups were significantly higher than those before treatment, whereas ESR, serum IgG, SLEDAI score, and TCMSSS score were significantly lower than those before treatment (*P*_hemoglobin_<0.001, *P*_ESR_<0.001, *P*_C3_=0.005, *P*_C4_=0.001, P_IgG_=0.012, *P*_SLEDAI_=0.045, *P*_TCMSSS_=0.006), with differences in these indicators between the observation and control groups (*P*<0.05) (Table 7).

**Table 7:** Comparison of clinical indicators, SLEDAI score, and TCMSSS score before and after treatment

***Indicators***	***Observation group (n=45)***		**t *value***	**P *value***	***Control group (n=62)***		**t *value***	**P *value***
	***Before treatment***	***After treatment***			***Before treatment***	***After treatment***		
Hemoglobin (g/L)	24.98±6.77	35.67±5.12^*^	−6.278	<0.001	26.16±6.73	29.50±5.81	−3.367	0.002
ESR (mm/h)	49.57±4.63	13.07±6.64^*^	22.686	<0.001	48.77±4.63	25.44±8.82	13.721	<0.001
Complement	0.55±0.18	1.03±0.20^*^	−8.370	<0.001	0.48±0.23	0.74±0.15	−2.426	0.022
C3(g/L)								
Complement	0.07±0.04	0.16±0.03^*^	−7.772	<0.001	0.09±0.03	0.14±0.02	−9.407	<0.001
C4(g/L)								
Serum IgG(g/L)	18.78±5.33	8.22±4.00^*^	7.897	<0.001	19.33±4.64	11.01±2.72	7.892	<0.001
SLEDAI score	16.55±2.78	8.72±2.57^*^	11.551	<0.001	19.02±3.87	7.97±1.55	14.280	<0.001
TCMSSS score	20.20±3.81	7.82±1.87^*^	16.305	<0.001	18.47±4.60	11.54±2.30	5.801	<0.001

Note: ESR indicates erythrocyte sedimentation rate

### Predictive values of IFN-γ and IL-4 before treatment

According to the efficacy, the patients were divided into the excellent efficacy group (Group A, n=48) and the poor efficacy group (Group B, n=59). IFN-γ and IL-4 before treatment were compared between the two groups.

**Fig. 1:**
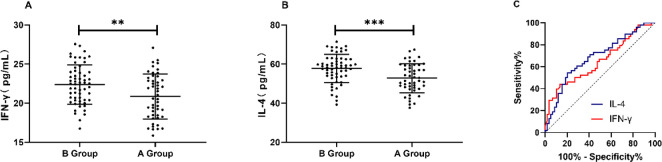
Predictive values of IFN-γ and IL-4 before treatment A: Changes in IFN-γ before and after treatment in the two groups. B: Changes in IL-4 before and after treatment in the two groups. C: ROC curves of IFN-γ and IL-4 for differentiating clinical efficacy

The results showed that their expressions before treatment in Group A were significantly lower than those in Group B (*P*<0.05). ROC curves showed that the areas under the ROC curves (AUCs) of IFN-γ and IL-4 were 0.649 and 0.684 respectively. The best specificity was 86.44%, the best sensitivity was 43.75%, and the Youden index was 30.19%, when the optimal cut-off value of IFN-γ was 19.83 pg/mL. The best specificity was 79.66%, the best sensitivity was 54.17%, and the Youden index was 33.83%, when the optimal cut-off value of IL-4 was 53.28 pg/mL. More details are shown in Fig. 1.

### Correlation analysis of IFN-γ and IL-4 with clinical indicators, SLEDAI score, and TCMSSS score after treatment

**Fig. 2:**
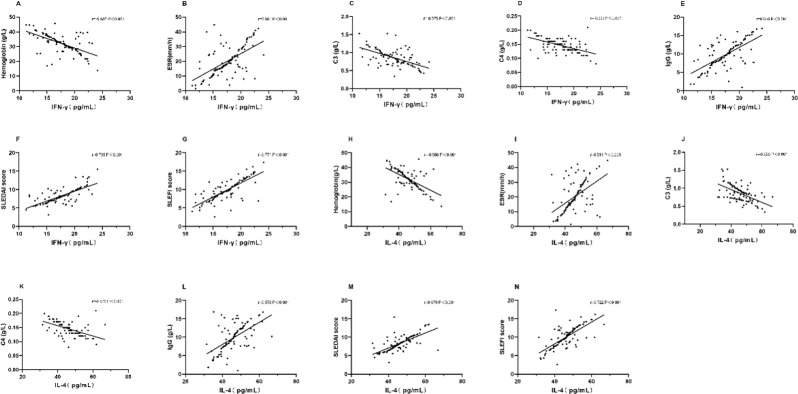
Correlation analysis of IFN-γ and IL-4 with clinical indicators, SLEDAI score, and TCMSSS score after treatment A. IFN-γ is negatively correlated with hemoglobin (r=−0.588, *P*<0.001). B. IFN-γ is positively correlated with ESR (r=0.591, *P*<0.001). C. IFN-γ is negatively correlated with complement C3 (r=−0.575, *P*<0.001). D. IFN-γ is negatively correlated with complement C4 (r=−0.553, *P*<0.001). E. IFN-γ is negatively correlated with serum IgG (r=0.646, *P*<0.001). F. IFN-γ is positively correlated with SLEDAI score (r=0.735, *P*<0.001). G. IFN-γ is positively correlated with TCMSSS score (r=0.771, *P*<0.001). H. IL-4 is negatively correlated with hemoglobin (r=−0.586, *P*<0.001). I. IL-4 is positively correlated with ESR (r=0.511, *P*<0.001). J. IL-4 is negatively correlated with complement C3 (r=−0.550, *P*<0.001). K. IL-4 is negatively correlated with complement C4 (r=−0.526, *P*<0.001). L. IL-4 is negatively correlated with serum IgG (r=0.578, *P*<0.001). M. IL-4 is positively correlated with SLEDAI score (r=0.679, *P*<0.001). N. IL-4 is positively correlated with TCMSSS score (r=0.722, *P*<0.001)

According to the Pearson correlation test, after treatment, IFN-γ and IL-4 were negatively correlated with hemoglobin, complement C3, and complement C4, but positively correlated with ESR, serum IgG, SLEDAI score, and TCMSSS score (*P*<0.05) (Fig. 2).

### Adverse reactions

After treatment, the observation group had 1 case of leukopenia, 2 cases of gastrointestinal discomfort, 0 cases of infection, and 2 cases of arrhythmia. The control group had 3 cases of leukopenia, 4 cases of gastrointestinal discomfort, 1 case of infection, and 3 cases of arrhythmia. There were no statistically significant differences between the two groups in the adverse reactions, and in the total incidence of adverse reactions (Table 8).

**Table 8:** Adverse reactions

***Groups***	***Leukopenia***	***Gastrointestinal discomfort***		***Infection***		***Arrhythmia***	***Total incidence***	
Control group (n=62)	3 (4.84)	4 (6.45)	1 (1.59)	3 (4.84)	11 (17.74)	3 (4.84)	4 (6.45)	1 (1.59)
Observation group (n=45)	1 (2.22)	2 (4.44)	0 (0.00)	2 (2.22)	5 (11.11)	1 (2.22)	2 (4.44)	0 (0.00)
X^2^	0.496	0.199	0.721	0.010	0.902	0.496	0.199	0.721
*P* value	0.481	0.656	0.396	0.924	0.342	0.481	0.656	0.396

## Discussion

In this study, the combination of cyclophosphamide, prednisolone, and Qing Shen Fang was used to treat patients with LN, and the efficacy improvement was observed. In a study, patients with LN were respectively treated with mycophenolate combined with prednisolone and cyclophosphamide combined with prednisolone. After treatment, the patients’ conditions significantly improved, their complements C3 and C4 in serum significantly increased, and their SLEDAI scores significantly improved (14).

The results of this study showed that efficacy improvement in the observation group was significantly better than that in the control group; the clinical indicators, SLEDAI score, and TCMSSS score in the two groups were significantly improved after treatment. These findings indicate that the combination of cyclophosphamide, prednisolone, and Qing Shen Fang is significantly more effective than cyclophosphamide and prednisolone alone, possibly because of the complementary effect of the drugs in Qing Shen Fang. Turtle carapace and sweet wormwood herb reduce asthenia heat and nourish yin. Common anemarrhena and danshen root nourish yin for lowering fire and remove heat to cool blood. Baical skullcap root and Chinese goldthread rhizome clear heat and remove toxicity. Red peony root promotes blood circulation to remove blood stasis. Prepared rhizome of rehmannia and common macrocarpium fruit nourish yin and reinforce kidney. The combination of these drugs can promote blood circulation to remove blood stasis, nourish yin for lowering fire, and clear heat and remove toxicity, as well as reinforce liver and kidney. The above studies demonstrate that the combination of cyclophosphamide, prednisolone, and Qing Shen Fang can improve the conditions of patients with LN.

At present, there are few standards for predicting and evaluating the efficacy in the treatment of LN. SLEDAI is a widely used score for SLE (15), but its scoring methods are subjective and prone to errors. Therefore, a new evaluation index should be found to solve this problem. As an important effector molecule in the body, T cells are usually divided into Th1 and Th2 cytokines. Th1 cytokines that activate macrophages are closely related to cytotoxicity and organ-specific auto-immune diseases. Th2 cytokines promote the activation of B lymphocytes, induce a large number of pathogenic autoantibody complexes, and then cause damage to tissues and organs (16, 17).

In recent years, more and more studies have proved that Th1 and Th2 cytokines are differentially expressed in the serum of patients with LN (18). In this study, after treatment, the expressions of IFN-γ and IL-4 in the observation and control groups were significantly lower than those before treatment, and the expressions in the observation group were lower than those in the control group. IFN-γ is a Th1 cytokine and IL-4 is a Th2 cytokine. Their expressions in the serum of patients with LN significantly reduce after treatment (19), which is similar to the results of this study. This further proves that the combination of cyclophosphamide, prednisolone, and Qing Shen Fang is more effective than cyclophosphamide and prednisolone alone.

For further observing the values of IFN-γ and IL-4 for the clinical efficacy, their expressions were compared. The expressions before treatment in Group B were significantly higher than those in Group A. Secondly, the ROC curves were plotted. The AUCs of IFN-γ and IL-4 were >0.6, which indicates that the expressions of the two before treatment have predictive values for efficacy in the treatment of LN. Thirdly, correlations of IFN-γ and IL-4 with clinical indicators were observed. The expressions of IFN-γ and IL-4 were negatively correlated with hemoglobin, complement C3, and complement C4, but positively correlated with ESR, serum IgG, SLEDAI score, and TCMSSS score. Hemoglobin, complement C3, complement C4, ESR, serum IgG, SLEDAI score, and TCMSSS score are important markers for the evaluation of LN (20).

The correlation analysis showed that the expressions of IFN-γ and IL-4 are correlated with these indicators, highly correlated with SLEDAI and TCMSSS scores. This indicates that IFN-γ and IL-4 are expected to be potential indicators for the efficacy evaluation of patients with LN after treatment. According to the analysis of the adverse reactions, there was no statistically significant difference between the observation and control groups in the incidence of adverse reactions, which shows that the combination of cyclophosphamide, prednisolone, and Qing Shen Fang does not increase the side effects on the patients.

## Conclusion

The combination of cyclophosphamide, prednisolone, and Qing Shen Fang improves conditions of patients with LN and significantly reduces their IFN-γ and IL-4 levels. IFN-γ and IL-4 can be used as potential indicators for the efficacy prediction and evaluation of LN.

## Ethical considerations

Ethical issues (Including plagiarism, informed consent, misconduct, data fabrication and/or falsification, double publication and/or submission, redundancy, etc.) have been completely observed by the authors.
